# Finding the most suitable puncture site for intraosseous access in term and preterm neonates: an ultrasound-based anatomical pilot study

**DOI:** 10.1007/s00431-023-04972-8

**Published:** 2023-04-19

**Authors:** Eva M. Schwindt, Theresa Häcker, Reinhold Stockenhuber, Janina M. Patsch, Sarah N. Mehany, Angelika Berger, Jens C. Schwindt

**Affiliations:** 1 Division of Neonatology, Pediatric Intensive Care and Neuropediatrics, Department of Pediatrics and Adolescent Medicine, Comprehensive Center for Pediatrics, Medical University Vienna, Vienna, Austria; 2 STAR-SIMCharacters Training and Research, Vienna, Austria; 3 Division of General and Pediatric Radiology, Department of Biomedical Imaging and Image-Guided Therapy, Medical University Vienna, Vienna, Austria; 4 Vienna Bone and Growth Center, Vienna General Hospital and Medical University of Vienna, Vienna, Austria; 5 Neonatal Working Group, Austrian Resuscitation Council, Graz, Austria

**Keywords:** Intraosseous access, Neonatal resuscitation, Puncture site, Anatomical landmarks, Tibial dimensions, Newborns

## Abstract

The purpose of this prospective ultrasound-based pilot study was to identify the most suitable tibial puncture site for intraosseous (IO) access in term and preterm neonates, describe tibial dimensions at this site, and provide anatomical landmarks for rapid localization. We measured the tibial dimensions and distances to anatomical landmarks at puncture sites A (proximal: 10 mm distal to the tibial tuberosity; distal: 10 mm proximal to the malleolus medialis) and B (chosen by palpation of the pediatrician), in 40 newborns in four weight groups (< 1000 g; 1000–2000 g, 2000–3000 g, and 3000–4000 g). Sites were rejected if they fell short of the assumed safety distance to the tibial growth plate of 10 mm. If both A and B were rejected, puncture site C was determined sonographically at the maximum tibial diameter while maintaining the safety distance. Puncture site A violated the safety distance in 53% and 85% (proximally and distally, respectively) and puncture site B in 38% and 33%. In newborns weighing 3000–4000 g, at median (IQR), the most suitable puncture site at the proximal tibia was 13.0 mm (12.0–15.8) distal to the tuberosity and 6.0 mm (4.0–8.0) medial to the anterior rim of the tibia. The median (IQR) diameters at this site were 8.3 mm (7.9–9.1) (transverse) and 9.2 mm (8.9–9.8) (anterior–posterior). The diameters increased significantly with increasing weight.

*  Conclusion:* This study adds concise, practical information on the implementation of IO access in neonatal patients: the tibial dimensions in newborns in four different weight groups and initial data on anatomical landmarks to easily locate the IO puncture site. The results may help implement IO access in newborns more safely.

**Table:** 

**What is Known:**
• *Intraosseous access is a feasible option for emergency administration of vital drugs and fluids in newborns undergoing resuscitation when an umbilical venous catheter is impossible to place.*
• *Severe complications of IO access due to malpositioned IO needles have been reported in neonates.*
**What is New:**
• *This study reports the most suitable tibial puncture sites for IO access and the tibial dimensions, in newborns of four weight groups.*
• *The results can help to implement safe IO access in newborns.*

## Introduction

During the rare event of newborn resuscitation, in addition to effective ventilation of the lung and chest compressions, venous access must be established rapidly to administer lifesaving drugs. In 2021, the guidelines for Newborn Life Support of the European Resuscitation Council included intraosseous (IO) access as a reasonable alternative to the primary method via umbilical vein catheters for emergency vascular access [1].

In pediatric patients aged less than 1 year, the recommended tibial puncture sites are 10 to 20 mm (or two finger-widths) distal to the tibial tuberosity at the proximal tibia and 10 to 20 mm proximal to the malleolus medialis at the distal tibia. This relatively broad recommendation was suggested to be adjusted to 10-mm distance at the proximal tibia for the age group of newborns [2–4]. However, the optimal puncture site in newborns has been rarely studied [3, 5–7].

To date, several studies and case reports describe the successful use of IO access in newborns [8–13]. However, severe complications of IO access due to malpositioned IO needles have been reported, including paravasation, bone fracture, and local infections [4, 5, 14, 15]. To avoid malposition and associated complications of IO access, it is important to puncture the tibia at its widest diameter to place the needle as safely as possible into the bone marrow cavity [5, 12]. In addition, puncture sites should be at a safe distance from the epiphyseal growth plate to reduce the risk of long-term sequelae such as growth retardation.

The aims of our study were to (1) identify the most suitable puncture site at the proximal and distal tibia for IO access in term and preterm neonates, (2) define distances to anatomical landmarks to easily locate the puncture site, and (3) provide the dimensions of the tibial bone at these sites.

## Methods

### Patients

As there is limited knowledge about the exact tibial bone growth rate at the IO puncture site in different gestational age or body weight groups [16, 17], this study was planned as a prospective pilot study. We included 40 term and preterm infants who were under postnatal care at the Department of Pediatrics and Adolescent Medicine of the Medical University of Vienna between June 2020 and April 2021. For each patient, we recorded body weight, body length, sex, gestational age (GA) at birth, and the chronological day of life (Table 1). The patients were divided into four weight groups (< 1000 g, 1000–2000 g, 2000–3000 g, and 3000–4000 g) with ten subjects each. The exclusion criteria were growth retardation (birth weight below the tenth percentile), other pathologies with potentially impaired bone geometry, morphology, and growth, skeletal dysplasias, musculoskeletal birth defects, malformations, or postnatal age of more than 14 days (to avoid any postnatal bias).

**Table 1:** Patient demographics across weight groups. Gestational age (GA), birth weight, length and age are given as mean with standard deviation (SD)

**Group**	GA (SD)*	**Birth weight (g)**	**Length (cm)**	**Age (d)**	**Female (%)**
** < 1000 g**	25 wk (± 9 d)	786 (± 111)	33.1 (± 1.2)	7 (± 3)	30
**1000–2000 g**	30 wk (± 14 d)	1455 (± 305)	40.7 (± 2.9)	6 (± 3)	10
**2000–3000 g**	36 wk (± 15 d)	2556 (± 300)	47.7 (± 2.7)	2 (± 2)	40
**3000–4000 g**	39 wk (± 8 d)	3525 (± 252)	50.9 (± 2.4)	4 (± 4)	90

*The mean value for GA refers to the week of pregnancy, the standard deviation to the number of days

**Fig. 1:**
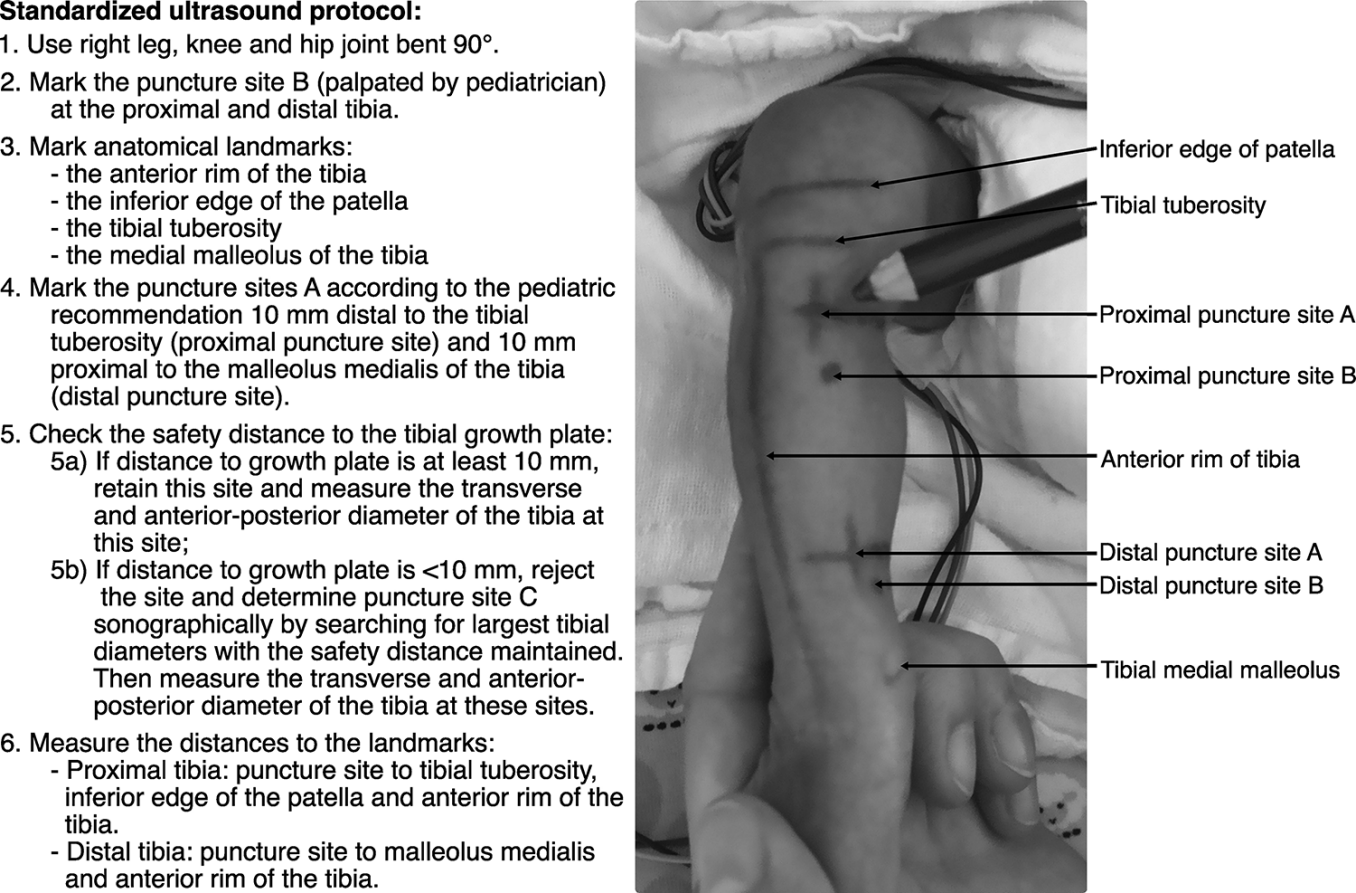
Illustrative example of the ultrasound protocol and markings in a preterm neonate, gestational age 30 + 3, of weight group 1000–2000 g

After obtaining written informed consent from the parents, the tibia of the newborn was measured using a specially developed standardized ultrasound protocol (Fig. 1), and, in addition, the tibial dimensions were measured via ultrasound images.

### Ultrasound protocol

All measurements were performed by two study team members (SM or TH) using a mobile ultrasound device (GE LOGIQ S8, General Electric Company, Boston, USA) and a linear probe (GE L8-18i-D, 8–18 MHz).

The aim was to identify the most suitable puncture site with the largest transverse (coronal) and anterior–posterior (sagittal) tibial diameters and at least 10-mm safety distance from the tibial growth plate (to avoid accidental injury to the growth plate).

We measured the proximal and distal tibial IO puncture sites according to a standardized acquisition protocol (Fig. 1) and determined the following puncture sites:

- Puncture site A: 10 mm distal to the tibial tuberosity, 10 mm proximal to the medial malleolus of the tibia (according to the lower border of the pediatric recommendation).
- Puncture site B: chosen by a pediatrician by palpation and subjective assessment.
- Puncture site C: sonographically determined in case puncture sites A and B violated the safe distance of 10 mm from the tibial growth plate.

One of the three puncture sites was designated as “most suitable” and used for further calculation; if either A or B met the safety distance, this site was used as “most suitable.” If A and B both met the safety distance, the site with the larger tibial diameter was considered. If neither A nor B met the safety distance, C was used. Measurements that exceeded 15-mm distance from the growth plate were further omitted accounting for the fact that the tibial diameter tapers with increasing distance from the growth plate resulting in smaller diameters.

The tibial tuberosity, inferior edge of the patella, anterior rim of the tibia, and medial malleolus were used as anatomical landmarks. The distance between the most suitable IO puncture site and these landmarks was measured on the skin surface.

**Fig. 2:**
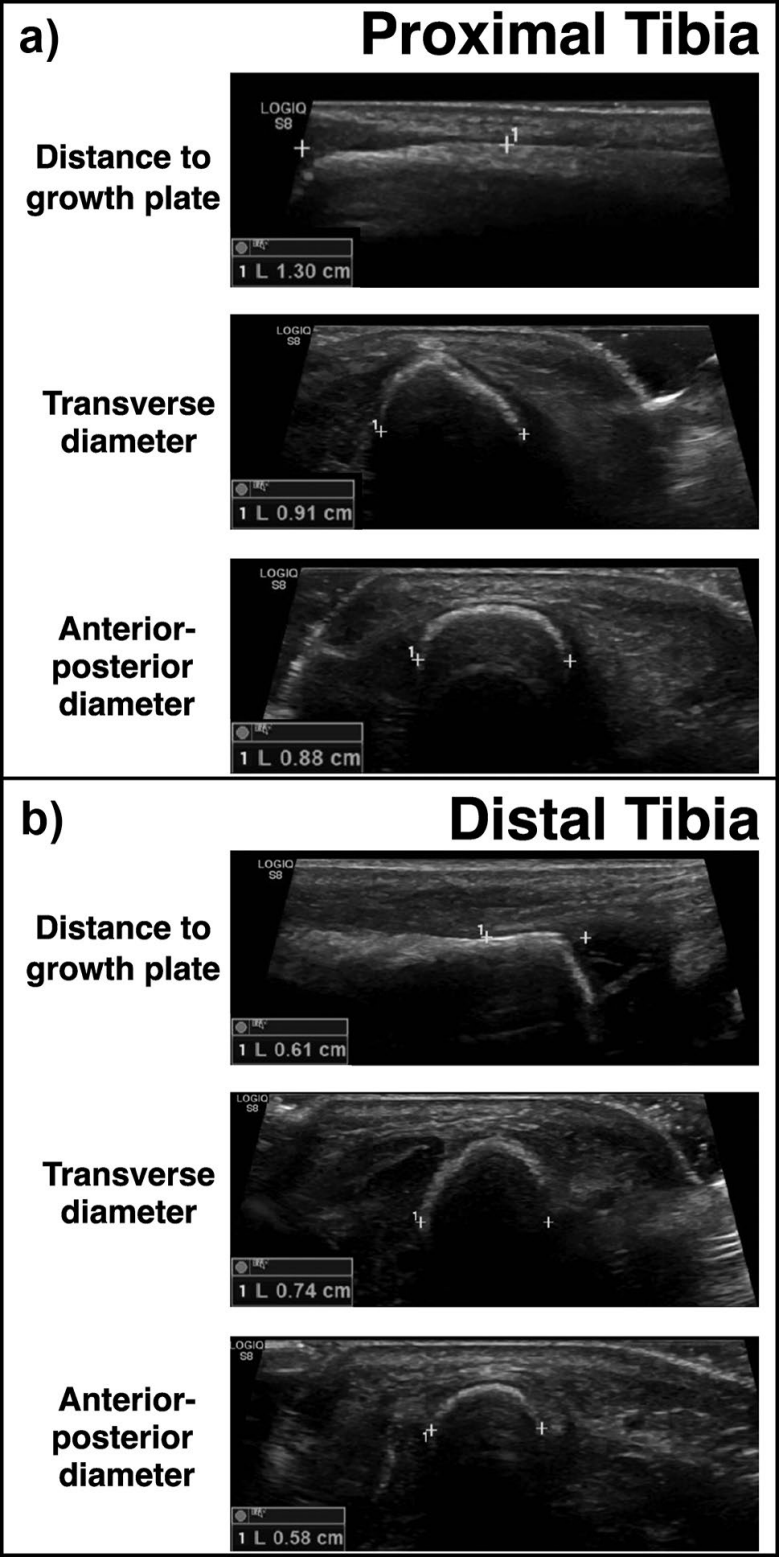
Illustrative example of ultrasound measurement in a term infant with a body weight of 3195 g at the **a**) proximal tibia and **b**) distal tibia

Tibial diameters were determined using ultrasonography (Fig. 2). The transverse and anteroposterior diameters were assessed for all A and B puncture sites that were retained (i.e., with the safety distance maintained) and for all C puncture sites.

### Statistical analysis

Statistical analysis was performed using R-Studio v4.1.2 (R Core Group, Austria), employing non-parametric Kruskal–Wallis rank-sum tests. Post hoc analyses for pairwise comparisons (Conover-Iman’s test) among the weight groups were performed with Bonferroni correction for multiple comparisons if the null-hypothesis, i.e., “all weight groups are equal,” was rejected by the Kruskal–Wallis test (*p* < 0.05).

This study was approved by the Ethics Committee of the Medical University of Vienna (EC code 1887/2018).

## Results

In total, data of 40 term and preterm infants (17 females and 23 males) were collected in this study, with ten subjects in each body weight group. The patient demographics are shown in Table 1. On an average, sonographic examination was performed on day 5 (days 1–13) of life.

### Determination of most suitable puncture sites

Puncture site A (10 mm distal to the tibial tuberosity, 10 mm proximal to the malleolus medialis) violated the safety distance to the growth plate in 53% and 85% of cases, at the proximal and distal tibia, respectively. Puncture site B (subjective assessment by pediatricians) violated the safety distance by 38% and 33% (proximal and distal, respectively). In 23% of cases, the safety distance was violated at both puncture sites A and B at the proximal tibia and in 38% of cases at the distal tibia. In these cases, puncture site C was identified using ultrasonography.

**Fig. 3:**
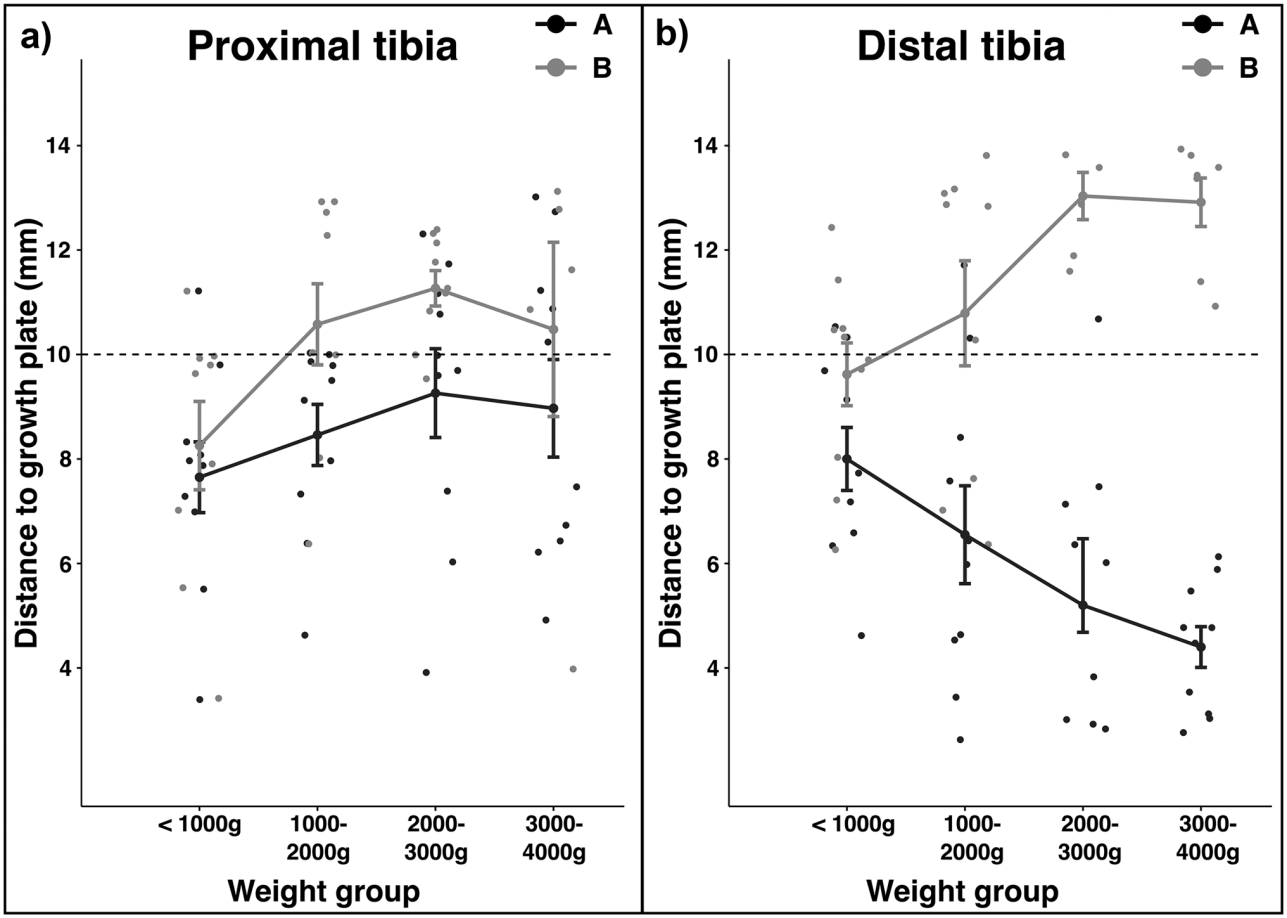
Measured distances of **A** and **B** marks to the growth plate at the **a**) proximal tibia and **b**) distal tibia. Each dot represents one measurement. The lineplots represent means (± 1 se) per group and visualize the differences between age groups for suggested (A, black) and pediatrician-determined (B, grey) puncture sites. The scattered line represents the safety distance of 10 mm. Indications are in mm (standard deviation)

The number of puncture sites A and B that met the safety distance, as well as the distance to the growth plate, increased with body weight, exempting puncture site A at the distal tibia, where the opposite trend was observed (Fig. 3).

### Anatomical landmarks

Anatomical landmarks can be used as guidance to locate the most suitable puncture sites. In the weight group with infants of 3000–4000 g body weight, at a median (interquartile range, IQR), the most suitable puncture site was located 13.0 mm (12.0–15.8) distally from the tibial tuberosity and 6.0 mm (4.0–8.0) medially from the anterior rim of the tibia. Details for the other weight groups and distal tibia as well as statistics are given in Table 2. The median (IQR) distance from the lower edge of the patella to the most suitable puncture site in the 3000–4000 g weight group was 21.0 mm (18.8–27.3) (Table 2).

**Table 2:** Median (IQR) values and statistics of the measured distances to selected landmarks from the most practicable proximal and distal puncture site, and median (IQR) values of tibial diameters at most practicable puncture sites. Indication in mm. Kruskal–Wallis chi-squared statistics (χ2) and significance levels (*p < .05; **p < .01; ***p < .001) are given in the rightmost column. Uppercase letters next to values indicate results of pairwise comparisons in compact letter display.

	**Measurement**	** < 1000 g**	**1000–2000 g**	**2000–3000 g**	**3000–4000 g**	**Statistics**
**Proximal**	Distance to patella (lower edge)	20.5 (20.0–22.0)	20.5 (19.3–22.0)	20.0 (20.0–24.3)	21.0 (18.8–27.3)	*χ*^2^ = 0.44 | ns
	Distance to tibial tuberosity	12.5 (11.0–14.5)	11.5 (10.0–13.8)	11.0 (9.25–14.5)	13.0 (12.0–15.8)	*χ*^2^ = 1.87 | ns
	Distance to anterior rim of tibia	3.5 (3.0–5.8)	5.0 (4.0–5.0)	6.5 (5.0–8.75)	6.0 (4.0–8.0)	*χ*^2^ = 6.56 | ns
	Transverse diameter	4.8 (4.3–5.0)A	6.4 (5.4–7.3)B	7.3 (6.8–7.7)BC	8.3 (7.9–9.1)C	*χ*^2^ = 24.57 |***
	Anteroposterior diameter	5.2 (5.1–5.8)A	7.1 (6.5–7.8)B	8.8 (8.1–9.4)C	9.2 (8.9–9.8)C	*χ*^2^ = 26.02 |***
**Distal**	Distance to malleolus medialis	13.0 (10.8–14.5)A	15.0 (14.0–16.0)A	15.0 (13.3–17.8)A	19.0 (18.0–20.0)B	*χ*^2^ = 18.57 |***
	Distance to anterior rim of tibia	5.5 (4.0–6.0)A	4.5 (4.0–6.5)A	7.5 (5.3–11.5)AB	10.0 (7.8–11.5)B	*χ*^2^ = 14.78 |**
	Transverse diameter	4.4 (4.2–4.8)A	5.4 (5.2–5.7)B	6.4 (6.2–6.7)C	6.8 (5.7–7.7)C	*χ*^2^ = 25.87 |***
	Anteroposterior diameter	4.3 (4.2–4.4)A	10.2 (6.6–10.5)B	10.6 (7.3–11.5)B	10.8 (10.1–11.6)B	*χ*^2^ = 22.50 |***

Values not sharing any letters are significantly different by the Conover-Iman test (*p* < .05)

At the proximal tibia, there was no significant difference in the distances from landmarks to puncture sites according to weight groups (distance to tibial tuberosity, *p* = 0.60; distance to lower edge of patella, *p* = 0.93). At the distal tibia, the distance from the landmarks malleolus medialis and anterior tibial rim to the most suitable puncture site was dependent on the weight group (*p* < 0.001 and *p* = 0.002), with larger landmark distances to the puncture site in groups with higher body weight.

### Tibial dimensions

**Fig. 4:**
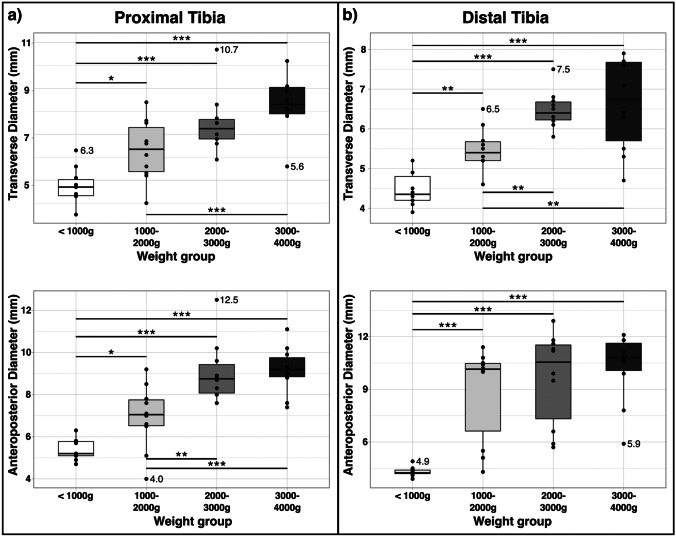
Boxplot of tibial diameters at the optimal puncture sites for four weight groups at the **a)** proximal tibia and **b**) distal tibia. Significant pairwise differences are marked by asterisks (*p < 0.05; **p < 0.01; ***p < 0.001). Outliers are represented as dots with numeric values. Indication in mm

For infants in the 3000–4000 g weight group, the median (IQR) transverse diameter at the most suitable puncture site of the proximal tibia was 8.3 mm (7.9–9.1) and the anterior–posterior diameter was 9.2 mm (8.9–9.8). At the distal tibia, we measured a median (IQR) transverse and anterior–posterior diameter of 6.8 mm (5.7–7.7) and 10.8 mm (10.1–11.6), respectively. Details of all weight groups are shown in Table 2 and Fig. 4. The transverse and anteroposterior diameters were significantly larger in the higher weight classes, both proximally and distally. Although the diameters tended to be larger at the proximal tibia, there was no significant difference in tibial diameters between the proximal and distal puncture sites.

## Discussion

The aims of this ultrasound-based pilot study were to (1) determine the most suitable puncture site for IO access in the tibia of term and preterm newborns, (2) identify anatomical landmarks to determine the most suitable puncture site, and (3) measure the tibial dimensions at the IO puncture site.

### Determination of most suitable puncture sites

To achieve high success rates in IO implementation and avoid complications, it is essential to accurately determine and locate the most suitable IO puncture site with the largest possible dimensions and diameters [5, 12]. However, to date, few studies have focused on the puncture site for IO access in newborns [2, 3, 7]. Consequently, recommendations for the suggested IO puncture site in the pediatric age group have been applied to neonates. These are 10–20 mm, corresponding to two finger-widths, distal to the tibial tuberosity for the proximal puncture site [4, 18, 19] and 10–20 mm proximal to the malleolus medialis for the distal puncture site [4].

In this study, to avoid accidental injury to the growth plate, we assumed the safe distance between the growth plate and the IO puncture site to be 10 mm. Interestingly, when following the lower limit of the aforementioned pediatric recommendation (10 mm distal to the tibial tuberosity, proximal to the malleolus medialis, respectively), this safety distance was often not met (in 53% of cases at the proximal tibia and 85% at the distal tibia). This was similar for puncture site B, which was subjectively chosen by the pediatrician (38% at the proximal tibia and 33% at the distal tibia). This finding is in contrast to that of Boon et al. [2], who found no injury to the tibial growth plate when performing IO access 10 mm distal to the tibial tuberosity.

However, it is important to note that the 10-mm safety distance to the growth plate was chosen as a purely pragmatic approach in the design of this study. To the best of our knowledge, there is no clear recommendation as to how large the safety distance to the growth plate should be. There are no clear data on the long-term consequences in cases of accidental perforation of the growth plate. Claudet et al. [20] found no difference in the growth of the tibia in children (aged 0.5–108 months) after the implementation of IO access. However, they did not specifically assess perforation of the growth plate in their study; hence, the long-term consequences of an actual growth plate injury remain elusive. There seem to be two possible conclusions: either the growth plate is indeed sometimes violated when implementing IO access in newborns, but due to the rarity of these events and the lack of clinical studies, no consequences have yet been noticed. Or alternatively, there is no consequence when the growth plate is violated, which means that the IO access could also be implemented further proximally, where the bone is wider, and thus, the probability of a correct needle position is increased. Long-term studies are clearly needed to answer this question.

### Anatomical landmarks

IO access is an extremely rare event even for experienced neonatologists. Therefore, it may be difficult to find the most suitable puncture site, especially in demanding, life-threatening emergencies. Specific anatomical landmarks to facilitate the localization of IO puncture sites would be beneficial; however, to our knowledge, so far, there is no study that has yet described clear landmarks. Therefore, we intended to provide initial data to fill this gap.

According to the results obtained in this pilot study, in a newborn with a body weight of 3000–4000 g, a measured median distance of 13.0 mm distal to the tibial tuberosity and 6.0 mm medial to the anterior rim of the tibia would lead to the most suitable puncture site for IO access at the proximal tibia. In addition, we provided data for newborns and preterms with lower body weights down to less than 1000 g. Interestingly, at the proximal tibia, these distances were similar across all weight groups and did not differ significantly in the weight groups. Certainly, these preliminary findings must be confirmed in future studies. Also, it must be emphasized that there is not yet sufficient data, and therefore, no recommendations can be made for the use of IO access in preterm infants. Manufacturers of IO needles also recommend a minimum weight for the establishment of IO access in newborns and preterm infants, which should be taken into account by providers.

It is noteworthy that, depending on the amount of fat tissue, palpability and clear identification of the tibial tuberosity were difficult in some cases. In addition, the tuberosity presents itself more as a plateau when palpated than as a clear demarcation line, which impedes its precise localization. In contrast, in most cases, the lower edge of the patella was palpated as a clear line, and therefore might represent an easier to palpate alternative landmark to find the most suitable IO puncture site. Additionally, the distance of the lower edge of the patella to the most suitable puncture site showed relatively low variation in our results.

To our knowledge, this is the first study to provide clinical guidance for anatomical landmarks based on ultrasound validation to determine the most suitable IO access in newborns. Nonetheless, owing to the pilot nature of this study and the small sample size in each weight group, future studies are warranted to derive recommendations for optimal IO access based on distances to anatomical landmarks in term and preterm neonates.

### Tibial dimensions

For the successful implementation of IO access in newborns, it is important to be aware of the expansion of tibia at the puncture site in order to safely place the IO needle in the medullary cavity [6, 7]. Therefore, we measured the transverse and anteroposterior diameters at the most suitable puncture sites determined in this study.

As expected, large differences between the weight groups were found, such that the diameters at the puncture sites were larger with increasing weight of the infants. The mean (median) diameter of the proximal tibia at the most suitable puncture site was 8.4 mm (8.3) (transverse) and 9.2 mm (9.2) (anterior–posterior) in the 3000–4000 g weight group. These diameters are slightly larger than those reported by Suominen et al. (7.4 mm transverse and 7.7 mm anterior–posterior diameter, assessed by X-ray) [5] and Tonder et al. (7.1 mm transverse and 7.7 mm anterior–posterior) [3], and smaller than those reported by Eifinger et al., who found an average diameter of 12.0 mm at the proximal tibia [7]. These differences may be a result of the selected measurement technique (i.e., X-ray or computer tomography vs. ultrasound) but also, compared to Eifinger et al., the site of measurement differed. While they measured the diameter at the level of the fibular head and the tibial tuberosity [7], we chose sites at least 10 mm below the tuberosity (safety distance). This could explain the smaller diameters of the tibia in our study, as the tibia is known to become narrower the further distally it is measured. The choice of the safety distance in this study clearly results in a limitation in the comparability with former studies. Nevertheless, from a clinical perspective, a deliberate puncture exactly at the level of the growth plate would presumably be avoided (at least as long as the long-term consequences after injury to the growth plate are not yet clear). Therefore, the results of this study may correspond more to the actual localization to be punctured for IO access in newborns, but this clearly has to be subject of future studies.

Also, future studies are warranted to analyze tibial diameters at different tibial heights to identify the optimal puncture sites as well as long-term consequences in case of injury to the growth plate.

### Limitations

We chose ultrasound as the method of investigation because it was most appropriate for term and preterm infants, some of whom required intensive care. Other methods, such as X-ray or computed tomography, may have yielded other or more precise results.

Furthermore, the relatively small number of patients in this pilot study does not allow us to establish normative values for the optimal site of intraosseous access in neonates. Nonetheless, it presents a valuable basis for further research on IO-puncture sites in neonates, which is essential for optimal and safe performance in emergency situations.

Finally, it must be emphasized that this study only investigated the tibia as an option for IO access, which seems to be the most commonly selected site for IO access in neonatal and pediatric resuscitation [4, 11], to date. Nonetheless, alternative puncture sites, such as the proximal humerus or distal femur, have been discussed [7] and should be further investigated.

## Conclusion

Knowledge of the optimal puncture site for IO access and its anatomical characteristics is crucial for the successful implementation of IO access in newborns. With this pilot study, we were able to present initial data for the creation of anatomical landmarks for rapid localization of the most suitable puncture site in neonates. In addition, we provided data on the dimensions of the tibia at the IO puncture site in the different weight groups. The findings of this study may help implement IO access more safely in newborns.

## Data Availability

The data sets used and/or analyzed in this study are available from the corresponding author upon request.

## Abbreviations

GA: Gestational age
IO: Intraosseous
IQR: Interquartile range
